# Algae–bacteria association inferred by 16S rDNA similarity in established microalgae cultures

**DOI:** 10.1002/mbo3.175

**Published:** 2014-05-05

**Authors:** Dagmar Schwenk, Liisa Nohynek, Heiko Rischer

**Affiliations:** VTT Technical Research Centre of FinlandEspoo, Finland

**Keywords:** Associated bacteria, characterization, diatom, green algae, marine bacteria, phycosphere

## Abstract

Forty cultivable, visually distinct bacterial cultures were isolated from four Baltic microalgal cultures *Chlorella pyrenoidosa, Scenedesmus obliquus, Isochrysis* sp., and *Nitzschia microcephala*, which have been maintained for several years in the laboratory. Bacterial isolates were characterized with respect to morphology, antibiotic susceptibility, and 16S ribosomal DNA sequence. A total of 17 unique bacterial strains, almost all belonging to one of three families, *Rhodobacteraceae, Rhizobiaceae*, and *Erythrobacteraceae*, were subsequently isolated. The majority of isolated bacteria belong to *Rhodobacteraceae*. Literature review revealed that close relatives of the bacteria isolated in this study are not only often found in marine environments associated with algae, but also in lakes, sediments, and soil. Some of them had been shown to interact with organisms in their surroundings. A Basic Local Alignment Search Tool study indicated that especially bacteria isolated from the *Isochrysis* sp. culture were highly similar to microalgae-associated bacteria. Two of those isolates, I1 and I6, belong to the Cytophaga–Flavobacterium–Bacteroides phylum, members of which are known to occur in close communities with microalgae. An UniFrac analysis revealed that the bacterial community of *Isochrysis* sp. significantly differs from the other three communities.

## Introduction

Species-specific interactions between bacteria and microalgae have been reported (Yoshinaga et al. 1995, 1997; Hasegawa et al. 2007), but remain underinvestigated due to their complexity. Interactions between bacteria and algae are essential in regulating algae accumulation and degradation of organic matter (Cole et al. 1988; Smith et al. 1995). The ability of algae-associated bacteria to flocculate algae increases sedimentation and removal of organic matter from the water column (Allison and Sutherland 1987). Especially bacterial communities associated with algal blooms play critical roles in carbon and nitrogen cycling through their influence on the formation and fate of dissolved organic matter (Cole et al. 1982; Azam and Ammerman 1984).

Studies on harmful algal blooms have revealed that marine bacteria are able to either stimulate phytoplankton growth (Ferrier et al. 2002) by releasing vitamins or growth factors (Haines and Guillard 1974; Maruyama et al. 1986), inhibit phytoplankton growth, or even destroy phytoplankton (Imai et al. 1995; Yoshinaga et al. 1997; Lovejoy et al. 1998; Amaro et al. 2005). More recent studies demonstrate that compositions of bacterial communities are not necessarily algae species-specific but depend to a high extent on the algae's extracellular products (Sapp et al. 2007). These results are in line with observations that diatom-attached bacteria are mainly algae species-specific while most free-living bacteria in the algae's surrounding are nonspecific (Grossart et al. 2005).

Exudates are among the key factors that influence the algal phycosphere, the zone directly surrounding algal cells. Within this zone, microbial activity is altered compared to that of the surrounding seawater (Bell and Mitchell 1972). Bacterial communities in phycospheres have been analyzed in several studies (Green et al. 2004; Jasti et al. 2005; Garces et al. 2007; Goecke et al. 2013). In particular, the growth-promoting effects for economically interesting algae have been extensively investigated (Gonzalez and Bashan 2000; Rivas et al. 2010). Usually, those studies are performed with algae samples taken directly from the sea. In most of the studies denaturing gradient gel electrophoresis was used (DGGE; Grossart et al. 2005; Rooney-Varga et al. 2005). With this method, large amounts of bacterial DNA can be identified, with the drawback that alive or dead bacteria cannot be distinguished. Furthermore, identified bacteria cannot be cultivated and characterized by classical microbiology methods.

Many microalgae cultures that are used in experiments, for example, for enhancing biomass production or lipid concentration for biofuel production, are cultures that are maintained for years in the respective laboratories. Bacterial communities in microalgae cultures do have an influence on metabolism of algae, and therefore it is necessary to study these bacteria in microalgae cultures regularly used in research or industrial applications.

In the present study, we examined bacteria coexisting in four microalgae cultures: *Chlorella pyrenoidosa, Scenedesmus obliquus, Isochrysis* sp., and *Nitzschia microcephala*. All cultures had been originally obtained from the Baltic Sea and were maintained for 5–8 years in standardized medium before this study was performed. Altogether we collected 40 bacterial isolates from these algal cultures and performed microbiological characterization and 16S ribosomal DNA (rDNA) analyses. With these experiments we gained a first insight into the phycosphere of cultivated microalgae.

## Experimental Procedures

### Microalgae cultures

The Baltic microalgae *C. pyrenoidosa* (strain code TV216), *S. obliquus* (strain code TVK-SOB-1), *Isochrysis* sp. (strain code TV-ISOCHR), and *N. microcephala* (strain code TV141) were obtained from the culture collection of the Tvärminne Zoological Station of the University of Helsinki, Finland, where they had been maintained for several years as liquid cultures (Hällfors and Hällfors 1992). By default, algal cultures were incubated in 250 mL Erlenmeyer flasks containing 50 mL of modified, sterilized f/2 medium (Guillard and Ryther 1962), denominated as T2 (Spilling et al. 2011). The cultures were incubated at 25°C with 90 rpm shaking and a 16:8 h light–dark cycle with 80 *μ*mol sec^−1^ m^−2^ illumination. The algal cultures were subcultured monthly.

### Isolation of associated bacteria

To isolate pure bacterial cultures from the algae, the four native algal cultures *C. pyrenoidosa, S. obliquus, Isochrysis* sp., and *N. microcephalia* were subcultured in fresh T2 medium and cultivated for 3 days. One milliliter of each culture was suspended in 9 mL of Marine Broth (BD Difco, Sparks, MD), and these suspensions were further diluted in the same way to 10^−3^ or 10^−4^. A 0.1 mL aliquot of each dilution was spread onto Marine Agar plates (Marine Agar 2216; BD Difco), and the plates were incubated according to the culturing temperature of the algae cultures at 25°C for 7 days. From each algal culture 10 single, visually distinct bacterial colonies were subcultured on fresh Marine Agar plates and incubated for 7 days. For long-term storage, the bacteria were conserved on beads (Protect Bacterial Preservers; Technical Service Consultants Ltd., Lancashire, UK) at −80°C. Isolated bacteria were labeled according to their algal culture origin, for example, C1–C10 for bacteria isolated from *Chlorella* culture, S1–S10 from *Scenedesmus* culture, and so on (Table 1).

**Table 1:** Characterization of bacterial isolates from Baltic microalgae *Nitzschia microcephala, Isochrysis* sp., *Chlorella pyrenoidosa*, and *Scenedesmus obliquus*

		16S rDNA analysis	Antibiotic susceptibility (antibiotic *μ*g/disk)									High similarity with
				
Bacteriacode	Morphology	Highest BLAST hit	% highest BLAST hit	Gentamycin 50	Streptomycin 25	Kanamycin 25	Rifampicin 5	Meropenem 5	Ticarcillin 50	Cefotaxime 50	Ampicillin 50	
*Nitzschia microcephala*												
N1^1^	Slender rod; beige	*Loktanella* sp.	100	1	1.8	–	4.1	1.8	4.1	5	3.3	N2, N4, N7
N2	Slender rod; beige	*Loktanella* sp.	100	1.5	1.8	–	4.1	1.8	4	5	3	N1, N4, N7
N3^1^	Slender rod; beige	*Loktanella* sp.	99	–	1.6	–	4^2^	2	4.2	5	3	N5^3^
N4	Slender rod; beige	*Loktanella* sp.	99	1.5	1.7	–	4.1	2.1	4.5	>5	2.6	N1, N2, N7
N5^1^	Slender rod; beige	*Loktanella* sp.	100	–	1.5	–	4	2.2	3.7	4.7	3.9	N3^3^
N6^1^	Plump pleomorphic rod; beige	*Loktanella* sp.	99	2.4	1.5	–	4.0^2^	3.3	3.6	5	2.6	N9
N7	Slender rod; beige	*Loktanella* sp.	100	1.5	2	–	3.8^2^	2	3.8	5	3	N1, N2, N4
N8^1^	Short rod; beige	*Loktanella* sp.	98	–	1.6	–	4	1.7	3.9	5	2.8	
N9	Plump pleomorphic rod; beige	*Agrobacterium* sp.	99	3^2^	1.6	–	4.2	2.1	4	5	3	N6
N10^1^	Very short rod, beige	*Loktanella* sp.	100	–	1.5	–	2.6	1.5	2.8	5	–	
*Isochrysis* sp.												
I1^1^	Pale slender rod; rust/orange	*Flexibacter* sp.	95	–	–	–	–	1.4	2.5	1.5	–	I2
I2	Pale slender rod; rust/orange	*Flexibacter* sp.	100	–	–	–	–	1.4	2.3	1.5	–	I1
I3^1^	Short rod; light pink	Rhodobacteraceae bacterium JAM-ALO110	99	3.8	2.2	–	3.5	2.9	4.5	>5	4.4	
I4^1^	Large pleomorphic rod; cream	*Seohaeicola saemankumensis* SD-15	98	3.7^2^	2.5^2^	1.5	4	3.8^2^	5	>5	4.3	
I5^1^	Large pleomorphic rod; cream	*Roseobacter* sp.	99	3.7^2^	2	1.7	4.5	4.5	>5	>5	>5	
I6^1^	Rods with cysts; cream	*Flexibacter* sp.	99	3	2	1.4	4	3.5	5	>5	4	
I7^1^	Short rod, red/orange	*Erythromicrobium* sp. B04	100	2.4	–	–	4.7	4	4.5	>5	3	I8, I9, I10
I8	Short rod, red/orange	*Erythromicrobium* sp. B04	100	2.3	–	–	4.5	3.7	4.5	>5	2.5	I7, I9, I10
I9	Short rod, red/orange	*Erythromicrobium* sp. B04	99	2.3	–	–	4.5	4.3	4.5	>5	3	I7, I8, I10
I10	Short rod, red/orange	*Erythromicrobium* sp. B04	100	2.2	–	–	4	4	4.5	>5	2.8	I7, I8, I9
*Chlorella pyrenoidosa*												
C1^1^	Short rod; pale	*Agrobacterium vitis* M63038	99	3.4	2.5	2	>4	>4	>5	>5	4.5	C3, C9, S8
C2^1^	Short rod; pale	*Agrobacterium vitis* M63038	96	3.5	2.4	1.6	3.6	>4	>5	>5	4	
C3	Short rod; pale	*Loktanella vestfoldensis* IMCC6033	100	3.4	2.2	1.6	3.4	4	>4.5	4.5	4.2	C1, C9, S8
C4^1^	Plump pleomorphic rod; pale	*Loktanella vestfoldensis* IMCC6033	99	2.8	1.5	–	4	3.2	3.8	>5	2.7	C6, C10
C5^1^	Plump pleomorphic rod; pale	*Agrobacterium vitis* M63038	98	2.5	1.4	–	4	3.6	4	>5	3.2	C7^3^, C8^3^
C6	Plump pleomorphic rod; pale	*Loktanella vestfoldensis* IMCC6033	100	2.5	1.4	–	4.3^2^	3.5	4	>5	3.5	C4, C10
C7^1^	Plump pleomorphic rod; pale	*Agrobacterium vitis* M63038	98	3	1.5	–	4.3	3.5	3.6	>5^2^	2.5	C5^3^, C8^3^
C8^1^	Plump pleomorphic rod; pale	*Agrobacterium vitis* M63038	98	2.7	1.6	–	4.5	3.2	4	>5	2.7	C5^3^, C7^3^
C9	Short rod; pale	*Loktanella vestfoldensis* IMCC6033	100	3.4	2.1	1.7	3.5	4	5	4.4	3.7	C1, C3, S8
C10	Plump pleomorphic rod; pale	*Loktanella vestfoldensis* IMCC6033	100	2.6	1.4	–	4.1	3.5	4	5.1	3.2	C4, C6
*Scenedesmus obliquus*												
S1^1^	Short rod; pale	*Agrobacterium vitis* M63038	98	3	2.2	1.5	3.2	2.6^4^	5	4.4	4	S2
S2	Short rod; pale	*Agrobacterium vitis* M63038	98	3.1	2.2	1.5	3	4	5	4.5	3.6	S1
S3^1^	Slender rod; beige	*Rhizobium* sp. W7	98	2.6	1.5	–	4.3	3.5	4	5^4^	3^4^	S4, S6, S9, S10
S4	Slender rod; beige	*Loktanella vestfoldensis* IMCC6033	100	2.6	1.5	–	4	3.4	4.2	5.3	3.2^4^	S3, S6, S9, S10
S5^1^	Short rod; pale	*Loktanella vestfoldensis* IMCC6033	100	2.8	1.5	2.4^2^	3.2	3.6^4^	>5	4.2	3.7	S7^3^
S6	Slender rod; beige	*Loktanella vestfoldensis* IMCC6033	99	2.8	1.5	–	4.2^2^	3^4^	4.2	5^4^	3^4^	S3, S4, S9, S10
S7^1^	Short rod; pale	*Loktanella vestfoldensis* IMCC6033	100	3.6	3	2	3	3.5^4^	>5	4.2	3.5	S5^3^
S8	Short rod; pale	*Loktanella vestfoldensis* IMCC6033	100	3.3	2.5	2.8^2^	3.2	3.8^4^	>5	4.4	4	C1, C3, C9
S9	Slender rod; beige	*Agrobacterium vitis* M63038	98	3	1.6	–	4.4	4^4^	4.7	5.5	3.5	S3, S4, S6, S10
S10	Slender rod; beige	*Loktanella vestfoldensis* IMCC6033	100	3	1.6	–	4.5^4^	4^4^	5	5.5	3.8	S3, S4, S6, S9

BLAST, Basic Local Alignment Search Tool.

1 Considered unique and used for the tree and UniFrac analysis.

2 Single colonies in inhibition zone.

3 Result of the maximum likelihood tree.

4 Inhibition zones were not totally clear.

### Characterization of marine bacteria

#### Morphological characterization

For morphological characterization, all 40 bacterial isolates were cultivated on Marine Agar 2216 plates as pure cultures. The colors of the bacterial colonies were recorded, and shape and Gram reaction of the bacterial cells were evaluated using a Polyvar microscope (Reichert-Jung, Wien, Austria) with 800× magnification. The Gram stain reaction of the bacteria was determined using a BD Gram Stain Kit (BD Diagnostics, Sparks, MD) according to the manufacturer's instructions. For the oxidase test a piece of filter paper was soaked in aqueous 1% *N,N,N′,N′*-tetramethyl-*p*-phenylenediaminedihydrochloride solution. Freshly grown bacteria were scraped from the plate and rubbed onto the filter paper. Development of blue color within 10 s was an indication of oxidase-positive isolates.

#### Antibiotic susceptibility

In order to test the antibiotic susceptibility of the isolated bacteria their pure cultures were cultivated in liquid medium (Marine Broth 2216; BD Difco) for 26–27 h at 25°C under shaking (120 rpm). Aliquots of 0.1 mL were spread evenly onto Marine Agar plates, allowing the liquid to permeate into the medium. Solutions of gentamycin, streptomycin, kanamycin, rifampicin, meropenem, ticarcillin, cefotaxim, and ampicillin (all antibiotics from Duchefa or Sigma, concentrations in Table 1) were prepared and 0.1 mL of each solution was added to separate disks (Antibiotika-Testblättchen; ø 12.7 mm, Schleicher & Schüll, Dassel, Germany). The disks were placed onto bacterial lawns and the plates were incubated at 25°C for 6 days. Clear inhibition zones were measured.

#### 16S ribosomal DNA (rDNA) analyses

For performing 16S ribosomal DNA (rDNA) analyses beads with preserved bacterial isolates were cultivated in liquid medium (Marine Broth 2216; BD Difco) for 26–27 h at 25°C under shaking (120 rpm). DNA was isolated by a modified cetyl trimethylammonium bromide protocol (Doyle and Doyle 1987). 16S rDNA (1450 bp) was amplified by PCR with Eubac27F and the 1492R primers according to Lane (1991).

**Figure 1:**
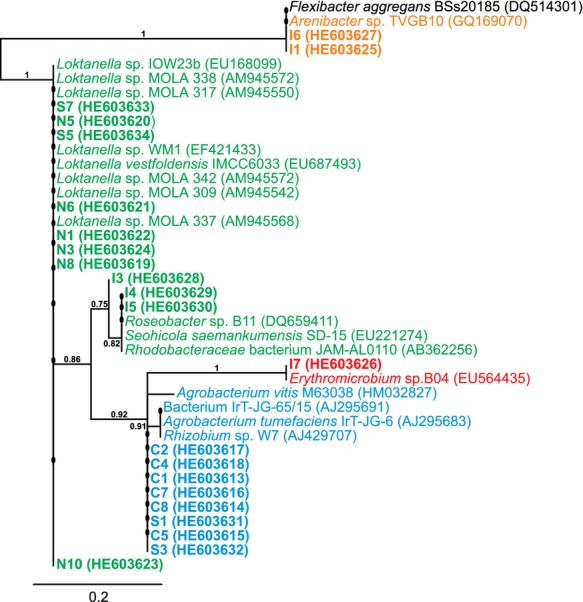
Maximum likelihood tree showing the relationships of the 22 marine bacteria (bold) isolated in this study and their closest relatives based on their 16S rDNA sequences. The DNA sequence accession numbers are shown in brackets. Strains belonging to different families are indicated by colors: *Flexibacteraceae* black, *Flavobacteraceae* orange, *Rhodobacteraceae* green, *Erythrobacteraceae* red, and *Rhizobiaceae* blue. Numbers indicate bootstrap values. The scale bar corresponds to 20 base substitutions per 100 nucleotide positions.

Resulting DNA sequences were compared to known DNA sequences in the NCBI database (Basic Local Alignment Search Tool, nucleotide blast). Sequences were aligned with MUSCLE 3.7 and a maximum likelihood tree was calculated using PhyML 3.0 aLRT and drawn by TreeDyn (Dereeper et al. 2010). All sequences used for phylogenetic analyses were deposited in the EMBL Nucleotide Sequence Database (for accession numbers see Fig. 1). To analyze the similarity of 16S rDNA sequences of the bacterial isolates with known sequences of phytoplankton-associated bacteria, 16S rDNA sequences were compared with known sequences in the NCBI database by using the Basic Local Alignment Search Tool (BLAST) function. All sequences with ≥99% similarity to our sequences were examined with respect to their origin. If the origin of the bacterial DNA sequence was a native algae culture, then this sequence was regarded as sequence from bacteria with algae association.

### Statistical evaluation

Statistical analyses were performed with the program SigmaPlot 11.0 (Systat Software, GmbH, Erkrath, Germany). A *t*-test (Mann–Whitney rank-sum test) was performed to test the pooled differences between the percentages of algae-associated sequences of bacteria isolated from *Isochrysis* and the three other algae cultures (*Nitzschia, Chlorella*, and *Scenedesmus*).

For comparing the four bacterial communities of *Isochrysis, Nitzschia, Chlorella*, and *Scenedesmus* unweighted UniFrac Significance tool (type of test: each environment individually) was used (Lozupone and Knight 2005). In general, UniFrac measures the distance between two environments in terms of the fraction of evolutionary history that separates the organisms in the two environments. Each algae culture was considered as an environment, each 16S rDNA sequence was assigned to one of the four environments. Calculations were performed with all 22 sequences from isolates considered unique (Table 1).

## Results

### Characterization of cultivable algae-associated bacteria

**Figure 2:**
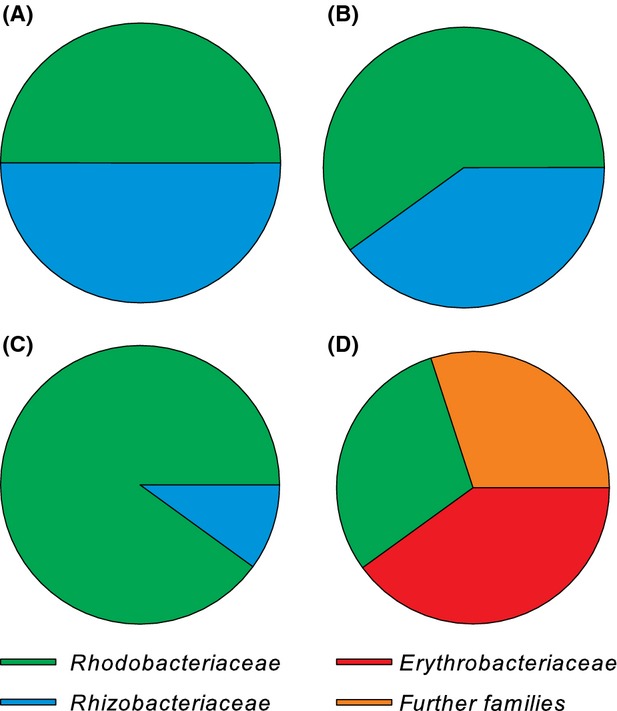
Families of bacteria isolated from microalgae cultures (A) *Chlorella pyrenoidosa*, (B) *Scenedesmus obliquus*, (C) *Nitzschia microcephala*, and (D) *Isochrysis* sp.

On the Marine medium plate of *N. microcephala, Isochrysis* sp., *C. pyrenoidosa*, and *S. obliquus* cultures 36, 386, 143, and 106 colonies, respectively, were observed. Ten separate bacterial colonies with different appearances were selected from each of the four microalgal cultures. All bacterial isolates were Gram-negative and oxidase-positive rods with variable susceptibility against selected antibiotics (Table 1). Furthermore, 16S rDNA analysis of all 40 bacterial isolates was performed, and on the basis of morphological characterization, antibiotic susceptibility, and comparison of 16S rDNA sequences to known sequences via BLAST, the collection of 40 bacterial isolates was clustered into 22 distinct groups of isolates. Results of the microbiological and genetic characterization are compiled in Table 1 and Figure 2. These sequence data have been also submitted to the EMBL database. The accession numbers are found in Figure 1.

The 16S rDNA sequences of these 22 strains, and those sequences from NCBI database with highest similarity to the sequences, were used for calculating the maximum likelihood tree (Fig. 1). Considering also the results of the tree, 17 of 22 isolated bacteria are unique. According to the combined results, 15 of the 17 bacterial strains were identified as belonging to the families *Rhodobacteraceae, Rhizobiaceae*, and *Erythrobacteraceae*, placed in the phylum Proteobacterium, and the remaining two isolates were localized in the Cytophaga–Flavobacterium–Bacteroides phylum (Fig. 1).

#### Rhodobacteracea

All bacterial strains isolated from the *Nitzschia* culture, coded from N1 to N10, showed very high 16S rDNA sequence similarity. Combining sequence similarity analysis with results from morphological characterization and the antibiotic susceptibility assays, six bacterial strains (N1, N3, N6, N8, and N10) were considered unique; while N5 is most probably similar to N3. Although all strains isolated from *Nitzschia* culture show a resistance against kanamycin, differences in antibiotic susceptibility were observed for gentamycin. No susceptibility for gentamycin was observed for strains N1 (similar to N2, N4, and N7) and N6 (similar to N9), while N3, N5, N8, and N10 were resistant against gentamycin. N1 and N6 were considered different strains because of their differences in microscopic appearance. Furthermore, the two strains showed a different antibiotic susceptibility against gentamycin and meropenem. The majority of isolates from *Nitzschia* showed typically slender rod morphology but N6 and N10 were plump and pleomorphic rods or very short rods, respectively. Additionally, the phylogenetic studies showed that strains S5 and S7 are most probably similar strains. This strain, isolated from *Scenedesmus*, clustered together with several strains isolated from the *Nitzschia* culture as well as with several *Loktanella* species offered by the NCBI database. Furthermore, their cell morphologies (plump pleomorphic/short rods) confirmed that, in addition to the *Nitzschia* isolates, also S5, S7, and C4 belong to the family of *Rhodobacteraceae*.

Bacterial strains I3, I4, and I5 isolated from *Isochrysis* sp. most probably belong to the family *Rhodobacteraceae*, too, but form a separate cluster. The 16S rDNA sequences of these isolates showed high similarity to the 16S rDNA of unclassified strains of *Rhodobacteraceae* (strain JAM-AL0110, AB362256), *Seohaeicola saemankumensis* and two *Roseobacter* sp. strains (B11 and DG869). In contrast to the other *Rhodobacteraceae* strains in this study, I4 and I5 were large pleomorphic rods without kanamycin sensitivity. Strain I3 differs from I4 and I5 by a short rod form, color, and sensitivity to kanamycin.

#### Rhizobiaceae

The second largest group was formed by the bacterial strains C1 (similar to C3, C9, and S8), C2, C5, C7, C8, isolated from the *C. pyrenoidosa* culture, together with S1 (similar to S2) and S3 (similar to S4, S6, S9, and S10), isolated from the *S. obliquus* culture. The maximum likelihood tree revealed that most probably C5 is similar to C7 and C8. Because of their high similarity with *Agrobacterium vitis* (strain M63038), *Rhizobium* sp. (strain W7), and *Agrobacterium tumefaciens* (strain IrT-JG-6), those bacteria might belong to the *Rhizobiaceae*. Cells of these strains varied between short rods (C1, C2, and S1) with sensitivity to kanamycin, slender rods (S3), and plump pleomorphic rods (C5, C7, and C8), both resistant to kanamycin.

#### Erythrobacteraceae

The bacterial strain I7 (similar to I8, I9, and I10) showed 100% similarity at the nucleotide level with *Erythromicrobium* sp. (phylum: Proteobacteria, order: Sphingomonadales). The colonies of I7 were colored from red to orange and the cells were short rods with resistance to streptomycin and kanamycin, which was not observed for any other isolated strains.

#### Further families

On the basis of phylogenetic analysis the strains I1 and I6 showed closest similarity to *Arenibacter* sp. (*Flavobacteraceae*) and *Flexibacter aggregans* (*Flexibacteraceae*), respectively, both being families of the Cytophaga–Flavobacterium–Bacteroides phylum. The slender rods of I1 (similar to I2) formed rust to orange-colored colonies and showed resistance to gentamycin, streptomycin, kanamycin, rifampicin, and ampicillin. I6 appeared as rods with cysts and was sensitive to all tested antibiotics.

### 16S rDNA sequence similarities of isolates from microalgae with known sequences of phytoplankton-associated bacteria

In order to quantify the association of isolated bacteria to algae, 16S rDNA sequences of the 22 unique bacteria were compared to known sequences in the NCBI database with BLAST function. All sequences with high similarity (≥99%) to 16S rDNA sequences from isolated bacteria were checked as to their origin. Table 2 shows the amount and percentage of those sequences with high similarity and an origin in a phytoplankton culture.

**Table 2:** Similarity of 16S rDNA of bacterial isolates from Baltic microalgae to sequences from phytoplankton-associated bacteria in NCBI database

Bacterial strain	Number of base position compared	Number of sequences with ≥99% similarity^1^	Number of sequences isolated from phytoplankton culture	Percentage of phytoplankton-associated sequences (%)
N1	608	100	6	6
N3	632	100	7	7
N5	504	100	7	7
N6	625	100	7	7
N8	298	0	0	0^2^
N10	138	100	3	3
I1	483	0	0	0^2^
I3	620	13	3	25
I4	527	0	0	0^2^
I5	569	14	3	25
I6	527	42	15	37
I7	564	12	3	27
C1	550	49	1	2
C2	522	0	0	0^2^
C4	710	31	8	28
C5	554	31	2	8
C7	572	58	2	4
C8	619	10	0	0
S1	635	9	0	0
S3	520	44	3	8
S5	638	100	7	7
S7	638	100	7	7

1 Maximal number of sequences shown in NCBI database with BLAST: 100 sequences.

2 Values are not used for further calculations.

Bacteria isolated from *Nitzschia, Chlorella*, and *Scenedesmus* cultures showed high similarity with on average 6.2%, 8.3%, and 5.5% (SE ± 0.8%, 5.0%, and 1.8%) of sequences with algae association. In contrast, 16S rDNA sequences of bacteria isolated from *Isochrysis* culture revealed a significant (*t*-test, *P* = 0.009) higher similarity than sequences isolated from all other microalgae cultures. Sequences of bacteria isolated from *Isochrysis* culture showed on average 28.5% (SE ± 2.8%) similarity to published sequences of algae-associated bacteria. Highest similarity with sequences of bacteria known to be associated with algae was identified for strain I6. Thirty-seven percent of the published sequences with similarity ≥99% revealed algae associations. Moreover, 16S rDNA sequence of strain C4 revealed a considerably high similarity (28%) to sequences of algae-associated bacteria as compared to other bacteria isolated from *Chlorella* culture.

### Comparison of the four bacterial communities using UniFrac

Based on an alignment of 22 16S rDNA sequences, the online tool UniFrac Significance indicated that the bacterial community of *Isochrysis* sp. differs significantly from the other communities (*P* < 0.01). This result is in line with the results of the comparison of the 16S rDNA sequences to sequences from phytoplankton-associated bacteria in NCBI database (Table 2). All other communities are not significantly different from each other (*P* = 0.72, *P* = 0.38, and *P* = 0.85 for *Nitzschia, Chlorella*, and *Scenedesmus*, respectively).

## Discussion

At the time of initiating a nonaxenic monoalgal culture it is conceivable that two groups of bacteria are present in the medium: Those that were coincidentally near the algae at the time of sampling or those that have some sort of relationship with the algae, ranging from symbiotic to pathogenic (Jasti et al. 2005). Regardless their connection, it is anticipated that bacteria are either favored or suppressed in the artificial situation and that a relatively balanced situation is reached within the years of continued subcultivation.

On the basis of microbiological characterization and sequence similarity, 40 visually distinct bacterial isolates, representing only a selection cultivable at these specific conditions, were assigned to four families, *Rhodobacteraceae, Rhizobiaceae, Erythrobacteraceae*, and *Flavobacteraceae*. Members of these families had been previously found worldwide in marine environments associated with algae as well as in lakes, sediments, and soil (Allgaier et al. 2003; Rooney-Varga et al. 2005; Penn et al. 2006; Jordan et al. 2007; Cho et al. 2008; Salka et al. 2008; Shigematsu et al. 2009; Xu et al. 2009).

### Rhodobacteraceae

The majority (55%) of the bacterial strains isolated in this study belong to the *Rhodobacteraceae* and show highest similarities regarding 16S rDNA with *Loktanella* sp. Although its presence in the Baltic Sea had been reported previously (Salka et al. 2008) *Loktanella*'s microalgae association has not yet been assessed. However, it has been recently shown that *Loktanella* sp. are more abundant on the surface of the green algae *Ulva australis* than in the surrounding sea water, which might owe to interaction of both species (Burke et al. 2011). It has been found that a *Loktanella* sp., isolated from the North Sea, releases a chemical compound showing high similarity to insect pheromones (Dickschat et al. 2005). In the present study, 16S rDNA sequences of three bacterial strains (I3, I4, and I5) clustered with the *Roseobacter* sp. B11 and two further *Rhodobacteraceae* sequences. *Roseobacter* species dominate among marine algae-associated bacteria and are engaged in intermittent symbiotic relations (reviewed by Buchan et al. 2005). *Phaeobacter gallaeciensis* belongs to the *Roseobacter* clade and produces antibacterial compounds in the biofilm of *U. australis*, thus preventing the growth of other bacteria on the algae (Ruiz-Ponte et al. 1998; Brinkhoff et al. 2004; Bruhn et al. 2005). It has been shown that breakdown compounds of the senescent algae stimulate *P. gallaeciensis* to produce potent and selective algicides, which ultimately kill the algae (Seyedsayamdost et al. 2011). Therefore, it is likely that the *Isochrysis* isolates I3, I4, and I5 also interact with *Isochrysis* sp. and probably with other microalgae and bacterial species as well.

### Erythrobacteriaceae

The bacterial strain I7 showed 100% 16S rDNA sequence similarity with *Erythromicrobium* sp. B04, which was isolated in the eastern Gotland Basin, Baltic Sea (Salka et al. 2008). Another study demonstrated the growth-inhibiting effects of *Erythromicrobium ramosum*, isolated from a bloom, on the cyanobacterium *Microcystis aeruginosa* (Shi et al. 2009). On the basis of 16S rDNA sequence analysis, many of the bacterial isolates described in this study are very close, or even identical with known bacterial species, such as strain I7 representing the cluster of isolates I7, I8, I9, and I10 from *Isochrysis* sp. These new isolates might even have potential in environmental biotechnology, because strains of *Erythromicrobium* are known to be highly tolerant toward toxic heavy-metal oxide tellurite by accumulating metallic crystals inside the cells (Yorkov et al. 1996). Also, several environmental bacterial isolates that are potentially useful for biodegradation and cleaning polluted sites are described as close relatives of *Erythromicrobium*, such as *Sphingomonas* (Nohynek et al. 1996; Tao et al. 2007). A detailed taxonomic study is still needed to identify these isolates, which would both increase information on associated cultures in marine environments and supplement the taxonomic scenery of marine bacteria.

### Rhizobiaceae

Members of the *Rhizobiaceae* were first isolated from soil (Conn 1938), later on also from marine sediments (Rüger and Höfle 1992; Süss et al. 2006; Jordan et al. 2007) and snow (Gonzalez-Toril et al. 2009). The genus *Agrobacterium* was affiliated to the genus *Rhizobium*, followed by renaming *Agrobacterium tumefaciens* to *Agrobacterium radiobacter* and *Agrobacterium rhizogenes* as *Rhizobium rhizogenes* (Young et al. 2001). However, in the present study the bacteria of this genus are named in accordance with the respective literature. In our study we isolated several bacteria with high similarity to *Agrobacterium* sp. at the 16S rDNA level. *Agrobacterium rhizogenes* (*Rhizobiaceae*) is known for its natural ability to insert bacterial DNA into the genome of higher plants (Tepfer 1984), a mechanism facilitating genetic engineering of plants. For a number of years *Agrobacterium* has also been used in the transformation of microalgae such as *Chlamydomonas reinhardii* (Kumar and Rajam 2007), *Haematococcus pluvialis* (Kathiresan et al. 2009), and *Nannochloropsis* sp. (Cha et al. 2011). Furthermore, a recent study revealed probiotic and growth-promoting properties of a *Rhizobium* sp. on the microalgae *Botryococcus braunii* (Rivas et al. 2010).

### Flavobacteriaceae

The 16S rDNA sequences of I1 and I6 showed highest similarity to *Arenibacter* sp. TVGB10 and *Flexibacter aggregans* BSs20185, both of which are members of the *Flavobacteriaceae*, belonging to the diverse Cytophaga–Flavobacterium–Bacteroides (CFB) phylum. *Flexibacter aggregans* is known as a fish pathogen belonging to a family of marine species with unclear taxonomic positions (Nedashkovskaya et al. 2005). *Arenibacter* was discovered in sandy sediment in the South China Sea and classified as a new genus of *Flavobacteriaceae* (Ivanova et al. 2001). Interestingly, further species of that genus, *Arenibacter palladensis* and *Arenibacter certesii*, were isolated from green algae and sea urchins (Nedashkovskaya et al. 2004, 2006; Urvantseva et al. 2006). Recently, *Arenibacter algicola* sp. nov., a polycyclic aromatic hydrocarbon degrading bacterium, was isolated from the marine diatom *Skeletonema costatum* which was obtained from a culture collection (Gutierrez et al. 2014). Despite their isolation from microalgae, interaction between algae and *Arenibacter* has not been described so far. Furthermore, Grossart and coworkers showed impressively that most bacteria which were associated with two diatoms belong to the CFB phylum (Grossart et al. 2005).

From all the bacterial isolates obtained in this study, those isolated from *Isochrysis* sp. culture showed highest similarity to known bacteria with algae association. This may indicate that bacteria that were found in the *Isochrysis* sp. culture have higher tendency of association or interference with the microalgae than other isolated bacteria. Results were confirmed by the UniFrac calculations and by the BLAST analysis (Table 2). Independently, both methods indicate that the bacterial community of *Isochrysis* sp. significantly differs from the three other communities.

In summary, the present study revealed that many of the bacterial strains isolated from Baltic microalgae cultures show high similarity to bacteria with confirmed algae association or even interaction. The isolated bacteria had survived several years of continuous algae maintenance in artificial medium, indicating that those bacteria benefit from their coexistence with microalgae. Especially the isolates I1 and I6 are likely to live associated with microalgae and might interfere with them. Possible interferences have not been well elucidated yet, and should be subject of future studies. For controlled and repeatable conditions in experiments with high-precision measurements of metabolic compounds such as lipids or glycosides the usage of axenic algae cultures should be considered working with microalgae. If axenic microalgae cultures are not available, it may be recommended to characterize the phycosphere of algae before performing other experimental work.

## References

[b1] Allgaier M, Uphoff H, Felske A, Wagner-Döbler I (2003). Aerobic anoxygenic photosynthesis in *Roseobacter* clade bacteria from diverse marine habitats. Appl. Environ. Microbiol.

[b2] Allison DG, Sutherland IW (1987). The role of exopolysaccherides in adhesion of freshwater bacteria. J. Gen. Microbiol.

[b3] Amaro AM, Fuentes MS, Ogalde SR, Venegas JA, Suárez-Isla BA (2005). Identification and characterization of potentially algal-lytic marine bacteria strongly associated with the toxic dinoflagellate *Alexandrium catenella*. J. Eukaryot. Microbiol.

[b4] Azam F, Fasham MJR, Ammerman JW (1984). Cycling of organic matter by bacterioplankton in pelagic marine ecosystems: microenvironmental considerations. Flows of energy and materials in marine ecosystems.

[b5] Bell W, Mitchell R (1972). Chemotactic and growth responses of marine bacteria to algal extracellular products. Biol. Bull.

[b6] Brinkhoff T, Bach G, Heidorn T, Liang L, Schlingloff A, Simon M (2004). Antibiotic production by a *Roseobacter* clade-affiliated species from the German Wadden Sea and its antagonistic effects on indigenous isolates. Appl. Environ. Microbiol.

[b7] Bruhn JB, Nielsen KF, Hjelm M, Hansen M, Bresciani J, Schulz S (2005). Ecology, inhibitory activity, and morphogenesis of a marine antagonistic bacterium belonging to the *Roseobacter* clade. Appl. Environ. Microbiol.

[b8] Buchan A, Gonzalez JM, Moran MA (2005). Overview of the marine *Roseobacter* lineage. Appl. Environ. Microbiol.

[b9] Burke C, Thomas T, Lewis M, Steinberg P, Kjelleberg S (2011). Composition, uniqueness and variability of the epiphytic bacterial community of the green alga *Ulva australis*. ISME J.

[b10] Cha T-S, Chen C-F, Yee W, Aziz A, Loh S-H (2011). Cinnamic acid, coumarin and vanillin: alternative phenolic compounds for efficient *Agrobacterium*-mediated transformation of the unicellular green alga, *Nannochloropsis* sp. J. Microbiol. Methods.

[b11] Cho Y, Hiramatsu K, Ogawa M, Omura T, Ishimaru T, Oshima Y (2008). Non-toxic and toxic subclones obtained from a toxic clonal culture of *Alexandrium tamarense* (Dinophyceae): toxicity and molecular biological feature. Harmful Algae.

[b12] Cole JJ, Likens GE, Strayer DL (1982). Photosynthetically produced dissolved organic carbon: an important carbon source for planktonic bacteria. Limnol. Oceanogr.

[b13] Cole JJ, Findlay S, Pace ML (1988). Bacterial production in fresh and saltwater: a cross-system overview. Mar. Ecol. Prog. Ser.

[b14] Conn H (1938). Taxonomic relationships of certain non-sporeforming rods in soil. J. Bacteriol.

[b15] Dereeper A, Audic S, Claverie JM, Blanc G (2010). BLAST-EXPLORER helps you building datasets for phylogenetic analysis. BMC Evol. Biol.

[b16] Dickschat JS, Wagner-Döbler I, Schulz S (2005). The chafer pheromone buibuilactone and ant pyrazines are also produced by marine bacteria. J. Chem. Ecol.

[b17] Doyle JJ, Doyle JL (1987). A rapid DNA isolation procedure for small quantities of fresh leaf tissue. Phytochem. Bull.

[b18] Ferrier M, Martin JL, Rooney-Varga JN (2002). Stimulation of *Alexandrium fundyense* growth by bacterial assemblages from the Bay of Fundy. J. Appl. Microbiol.

[b19] Garces E, Vila M, Rene A, Alonso-Saez L, Angles S, Luglie A (2007). Natural bacterioplankton assemblage composition during blooms of *Alexandrium* spp. (Dinophyceae) in NW Mediterranean coastal waters. Aquat. Microb. Ecol.

[b20] Goecke F, Thiel V, Wiese J, Labes A, Imhoff JF (2013). Algae as an important environment for bacteria – phylogenetic relationships among new bacterial species isolated from algae. Phycologia.

[b21] Gonzalez LE, Bashan Y (2000). Increased growth of the microalga *Chlorella vulgaris* when coimmobilized and cocultured in alginate beads with the plant-growth-promoting bacterium *Azospirillum brasilense*. Appl. Environ. Microbiol.

[b22] Gonzalez-Toril E, Amils R, Delmas RJ, Petit JR, Komarek J, Elster J (2009). Bacterial diversity of autotrophic enriched cultures from remote, glacial Antarctic, Alpine and Andean aerosol, snow and soil samples. Biogeosciences.

[b23] Green DH, Llewellyn LE, Negri AP, Blackburn SI, Bolch CJS (2004). Phylogenetic and functional diversity of the cultivable bacterial community associated with the paralytic shellfish poisoning dinoflagellate *Gymnodinium catenatum*. FEMS Microbiol. Ecol.

[b24] Grossart HP, Levold F, Allgaier M, Simon M, Brinkhoff T (2005). Marine diatom species harbour distinct bacterial communities. Environ. Microbiol.

[b25] Guillard R, Ryther J (1962). Studies of marine planktonic diatoms. I. *Cyclotella nana* Hustedt, and *Detonula confervacea* (Cleve) *Gran*. Can. J. Microbiol.

[b26] Gutierrez T, Rhodes G, Mishamandani S, Berry D, Whitman WB, Nichols PD (2014). Polycyclic aromatic hydrocarbon degradation of phytoplankton-associated *Arenibacter* spp. and description of *Arenibacter algicola* sp. nov., an aromatic hydrocarbon-degrading bacterium. Appl. Environ. Microbiol.

[b27] Haines KC, Guillard RRL (1974). Growth of vitamin B_12_-requiring marine diatoms in mixed laboratory cultures with vitamin B_12_-producing marine bacteria. J. Phycol.

[b28] Hällfors G, Hällfors S (1992). The Tvärminne collection of algal cultures. In Tvärminne Studies.

[b29] Hasegawa Y, Martin JL, Giewat MW, Rooney-Varga JN (2007). Microbial community diversity in the phycosphere of natural populations of the toxic alga, *Alexandrium fundyense*. Environ. Microbiol.

[b30] Imai I, Ishida Y, Sakaguchi K, Hata Y (1995). Algicidal marine bacteria isolated from northern Hiroshima Bay, Japan. Fish. Sci.

[b31] Ivanova EP, Nedashkovskaya OI, Chun J, Lysenko AM, Frolova GM, Svetashev VI (2001). *Arenibacter* gen. nov., new genus of the family *Flavobacteriaceae* and description of a new species, *Arenibacter latericius* sp. nov. Int. J. Syst. Evol. Microbiol.

[b32] Jasti S, Sieracki ME, Poulton NJ, Giewat MW, Rooney-Varga JN (2005). Phylogenetic diversity and specificity of bacteria closely associated with *Alexandrium* spp. and other phytoplankton. Appl. Environ. Microbiol.

[b33] Jordan EM, Thompson FL, Zhang X-H, Li Y, Vancanneyt M, Kroppenstedt RM (2007). *Sneathiella chinensis* gen. nov., sp. nov., a novel marine alphaproteobacterium isolated from coastal sediment in Qingdao, China. Int. J. Syst. Evol. Microbiol.

[b34] Kathiresan S, Chandrashekar A, Ravishankar GA, Sarada R (2009). *Agrobacterium*-mediated transformation in the green alga *Haematococcus pluvialis* (Chlorophyceae, Volvocales). J. Phycol.

[b35] Kumar SV, Rajam MV (2007). Induction of *Agrobacterium tumefaciens vir* genes by the green alga, *Chlamydomonas reinhardtii*. Curr. Sci.

[b36] Lane DJ (1991). Nucleic acid techniques in bacterial systematics.

[b37] Lovejoy C, Bowman JP, Hallegraeff GM (1998). Algicidal effects of a novel marine *Pseudoalteromonas* isolate (class Proteobacteria, gamma subdivision) on harmful algal bloom species of the genera *Chattonella*
*Gynodinium*, and *Heterosigma*. Appl. Environ. Microbiol.

[b38] Lozupone C, Knight R (2005). UniFrac: a new phylogenetic method for comparing microbial communities. Appl. Environ. Microbiol.

[b39] Maruyama A, Maeda M, Simidu U (1986). Occurrence of plant hormone (cytokinin)-producing bacteria in the sea. J. Appl. Microbiol.

[b40] Nedashkovskaya OI, Kim SB, Han SK, Lysenko AM, Mikhailov VV, Bae KS (2004). *Arenibacter certesii* sp. nov., a novel marine bacterium isolated from the green alga *Ulva fenestrata*. Int. J. Syst. Evol. Microbiol.

[b41] Nedashkovskaya OI, Kim SB, Lee DH, Lysenko AM, Shevchenko LS, Frolova GM (2005). *Roseivirga ehrenbergii* gen. nov., sp. nov., a novel marine bacterium of the phylum ‘Bacteroidetes’, isolated from the green alga *Ulva fenestrata*. Int. J. Syst. Evol. Microbiol.

[b42] Nedashkovskaya OI, Vancanneyt M, Cleenwerck I, Snauwaert C, Kim SB, Lysenko AM (2006). *Arenibacter palladensis* sp. nov., a novel marine bacterium isolated from the green alga *Ulva fenestrata*, and emended description of the genus *Arenibacter*. Int. J. Syst. Evol. Microbiol.

[b43] Nohynek L, Nurmiaho-Lassila E-L, Suhonen E, Busse HJ, Mohammadi M, Hantula J (1996). Description of chlorophenol-degrading *Pseudomonas* sp. strains KF1T, KF3, and NKF1 as a new species in the genus *Sphingomonas*
*Sphingomonas subarctica* sp. nov. Int. J. Syst. Bacteriol.

[b44] Penn K, Wu DY, Eisen JA, Ward N (2006). Characterization of bacterial communities associated with deep-sea corals on Gulf of Alaska seamounts. Appl. Environ. Microbiol.

[b45] Rivas MO, Vargas P, Riquelme CE (2010). Interactions of *Botryococcus braunii* cultures with bacterial biofilms. Microb. Ecol.

[b46] Rooney-Varga JN, Giewat MW, Savin MC, Sood S, LeGresley M, Martin JL (2005). Links between phytoplankton and bacterial community dynamics in a coastal marine environment. Microb. Ecol.

[b47] Rüger H-J, Höfle MG (1992). Marine star-shaped-aggregate-forming bacteria: *Agrobacterium atlanticum* sp. nov.; *Agrobacterium meteori* sp. nov.; *Agrobacterium ferrugineum* sp. nov., nom. rev.; *Agrobacterium gelatinovorum* sp. nov., nom. rev.; and *Agrobacterium stellulatum* sp. nov., nom. rev. Int. J. Syst. Bacteriol.

[b48] Ruiz-Ponte C, Cilia V, Lambert C, Nicolas JL (1998). *Roseobacter gallaeciensis* sp. nov., a new marine bacterium isolated from rearings and collectors of the scallop *Pecten maximus*. Int. J. Syst. Bacteriol.

[b49] Salka I, Moulisova V, Koblizek M, Jost G, Jurgens K, Labrenz M (2008). Abundance, depth distribution, and composition of aerobic bacteriochlorophyll *a*-producing bacteria in four basins of the central Baltic Sea. Appl. Environ. Microbiol.

[b50] Sapp M, Schwaderer AS, Wiltshire KH, Hoppe HG, Gerdts G, Wichels A (2007). Species-specific bacterial communities in the phycosphere of microalgae?. Microb. Ecol.

[b51] Seyedsayamdost MR, Case RJ, Kolter R, Clardy J (2011). The Jekyll-and-Hyde chemistry of *Phaeobacter gallaeciensis*. Nat. Chem.

[b52] Shi L, Cai Y, Li P, Yang H, Liu Z, Kong L (2009). Molecular identification of the colony-associated cultivable bacteria of the cyanobacterium *Microcystis aeruginosa* and their effects on algal growth. J. Freshwater Ecol.

[b53] Shigematsu T, Hayashi M, Kikuchi I, Ueno S, Masaki H, Fujii T (2009). A culture-dependent bacterial community structure analysis based on liquid cultivation and its application to a marine environment. FEMS Microbiol. Lett.

[b54] Smith DC, Steward GF, Long RA, Azam F (1995). Bacterial mediation of carbon fluxes during a diatom bloom in a mesocosm. Deep-Sea Res. Pt. II.

[b55] Spilling K, Seppälä J, Tamminen T (2011). Inducing autoflocculation in the diatom *Phaeodactylum tricornutum* through CO_2_ regulation. J. Appl. Phycol.

[b56] Süss J, Schubert K, Sass H, Cypionka H, Overmann J, Engelen B (2006). Widespread distribution and high abundance of *Rhizobium radiobacter* within Mediterranean subsurface sediments. Environ. Microbiol.

[b57] Tao X-Q, Lu G-N, Dang Z, Yang C, Yi X-Y (2007). A phenanthrene-degrading strain *Sphingomonas* sp. GY2B isolated from contaminated soils. Process Biochem.

[b58] Tepfer D (1984). Transformation of several species of higher plants by *Agrobacterium rhizogenes*: sexual transmission of the transformed genotype and phenotype. Cell.

[b59] Urvantseva AM, Bakunina IY, Nedashkovskaya OI, Kim SB, Zvyagintseva TN (2006). Distribution of intracellular fucoidan hydrolases among marine bacteria of the family *Flavobacteriaceae*. Appl. Biochem. Microbiol.

[b60] Xu X-W, Wu Y-H, Wang C-S, Wang X-G, Oren A, Wu M (2009). *Croceicoccus marinus* gen. nov., sp. nov., a yellow-pigmented bacterium from deep-sea sediment, and emended description of the family *Erythrobacteraceae*. Int. J. Syst. Evol. Microbiol.

[b61] Yorkov V, Jappe J, Vermeglio A (1996). Tellurite resistance and reduction by obligately aerobic photosynthetic bacteria. Appl. Environ. Microbiol.

[b62] Yoshinaga I, Kawai T, Takeuchi T, Ishida Y (1995). Distribution and fluctuation of bacteria inhibiting the growth of a marine red tide phytoplankton *Gymnodinium mikimotoi* in Tanabe Bay, Wakayama Pref., Japan. Fish. Sci.

[b63] Yoshinaga I, Kawai T, Ishida Y (1997). Analysis of algicidal ranges of the bacteria killing the marine dinoflagellate *Gymnodinium mikimotoi* isolated from Tanabe Bay, Wakayama Pref., Japan. Fish. Sci.

[b64] Young JM, Kuykendall LD, Martinez-Romero E, Kerr A, Sawada H (2001). A revision of *Rhizobium* Frank 1889, with an emended description of the genus, and the inclusion of all species of *Agrobacterium* Conn 1942 and *Allorhizobium undicola* de Lajudie, 1998 as new combinations: *Rhizobium radiobacter*
*R. rhizogenes*
*R. rubi*
*R. undicola* and *R. vitis*. Int. J. Syst. Evol. Microbiol.

